# Building bridges between Ayurveda and Modern Science

**DOI:** 10.4103/0974-7788.59943

**Published:** 2010

**Authors:** Sanjeev Rastogi

**Affiliations:** *Department of Kaya Chikitsa, State Ayurvedic College and Hospital, Tulsi Das Marg, Lucknow - 4, Department of Holistic Medicine, BMCRC, Vatsala Hospital, Tulsi Das Marg, Lucknow, India*

**Keywords:** Ayurveda, science, *tridosha*

## Abstract

The recent decade has witnessed many landmark observations, which have added to the scientific credentials of Ayurveda.It is however believed that instead of a retrospective approach of looking into the Ayurveda through the scientific reappraisals, a prospective approach through primary understanding of Ayurveda followed by a search into scientific linkage would be more appealing. This article brings the simplified yet scientific decoding of the core concepts of Ayurveda that form the framework of this ancient science of health.

## INTRODUCTION

The 21st century has marked the beginning of a new era, receptive to eastern healthcare philosophy through positive attitudes.[1] Epitomizing eastern philosophy for its concern to values related to health, Ayurveda is by and large considered to showcase traditional health care. Before the recent upsurge of traditional medicine in a global perspective, Ayurveda was persistently criticized for its ambiguity and philosophical tenets incomprehensible to occidental mind. This perception has led to disinterest in Ayurveda in the western world, which eventually and unfortunately has led the world to be deprived of many plausible advantages of traditional healthcare supportive to a total quality life (TQL).[2–5]

Contrary to the global scene, Ayurvedic schools in India consistently urge for scientific rooting of Ayurvedic principles. A connotation of linking science and Ayurveda is well perceived by Ayurvedic physicians in India, and schools like Banaras Hindu University (BHU) have pioneered this thought. What prevented this thought from becoming a reality? A poor scientific appraisal of Ayurvedic principles among Indian scientific community eventually resulted in a poor interdisciplinary collaboration between science and Ayurveda. This subsequently led to a premature demise of this thought until it has been resurrected recently with stronger evidence to rely on. Thinking of effective utilization of Ayurveda through a better understanding of its fundamentals is the recent realization. Fortunately, this realization has given rise to a spurt of an impulsive and argumentative pro-Ayurvedic generation as its eventuality.[6] Global dissemination of Ayurveda required its scientific presentation besides minimization of its semantic barriers helping it to become comprehensible to the people exogenous to it. Fortunately, a few among the new genre have prompted to sense this reality and stand up as intermediaries, speaking in a mutually understandable language and retrieving the link of Ayurveda to science while keeping its original flavor intact.[7,8]

## ORIGIN OF LIFE: FROM AYURVEDA TO THEORY OF BIOPOIESIS

How did life evolve on Earth? This is the question stirring the human mind all through the ages. Initial proposals of spontaneous generation soon transformed into *omne vivum ex ovo* (every living thing comes from a pre-existing living thing) before arriving finally at theory of biopoiesis. A biopoiesis theory ultimately proposed for a three-step transition between non-livings to living earth. These transitional stages are postulated as:

1. Origin of biological monomers; 2. Origin of biological polymers and 3. Evolution of cells from polymers.[9]

Revisiting the recent understanding of origin of life through an Ayurvedic viewpoint surprisingly explores a remarkable clarity and similarity of postulates.

Elaborating its postulates about origin of life, Ayurveda, fundamentally proposes a few dictums, which are equally re-phrasable to their modern contexts of origin of life. These dictums are:

1. *Nasato vidyate bhavo, na bhavo vidyate satam* (non- existence cannot give rise to existence) 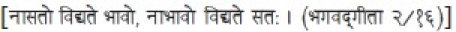 as an Ayurvedic version to *omne vivum ex ovo* (every living thing comes from a pre-existing living thing). This dictum argues for the omnipresence of formative substances of life since eternity.
2. Origin of life process began from *Avyakta* (invisible) and reached to *Vyakta* (visible) stage after passing through number of intermediaries.

Ayurveda proposes for an omnipresence of basic building blocks of life in the universe suggesting that beginning of synthesis is subject to the availability of optimal conditions. This justifies the life process to begin only a few billion years ago despite availability of the basic materials since eternity. Primitive earth was proposed to be characterized by *Sattva, Rajas* and *Tamas (Triguna)* symbolizing the physical properties prevalent to the primitive earth. *Sattva*, the foremost of *Triguna*, symbolizes the energy required for creation, *Rajas*, the second of *Triguna* symbolizes the particle movements and *Tamas* finally symbolizes inert material having a capacity to convert into new forms under the constant influence of *Sattva* and *Rajas*.

It is interesting to hypothesize a resemblance between *Sattva, Rajas* and *Tamas* to that of conditions available to primitive earth that acted decisively to begin the actual process of origin of life [Table 1].

**Table 1:** Classical attributes of Triguna and their hypothetical inference

Triguna	Quality	Hypothetical infere nce
Sattva	Pure, act as an stimulus to start the process and finally guides to the direction of reaction	Sunlight
Rajas	Movement	Movements of atoms or their components, initially randomized so non- creative but when directed, may lead to creation
Tamas	Inert basic elements, precursor to other substances when added under the influence of Sattva and Rajas	Basic raw material, which finally lead to the formation of organic structures.

Ayurveda argues for life beginning from invisible components. While passing through many intermediate stages these components reach a physically visible stage called as *Mahabhuta* (*maha = *big, *bhuta = *first form of existence). *Mahabhuta* are proposed to be the consolidation products of invisible formative units or *Tanmatra* (smallest possible component of *Mahabhuta* having similar properties, but invisible). Eventually, a *Mahabhuta* in turn happens to be a consolidation and combination product of various *Tanmatra* in a predetermined differential order. It is interesting to note that only five types of *Tanmatra* and consequently five *Mahabhuta* are described in Ayurveda. This numerological agreement upon the number of *Mahabhuta* is in accordance with the physically perceptible forms of matter, its absence and also with the state of energy acting in transition. The five *Mahabhuta* are also linked to the five sense organs as means of their perception [Table 2]. It is further interesting to note that Ayurveda has described the element of *Agni* (fire) as a *Mahabhuta* long before the proposition of energy and matter transitions by Einstein.

**Table 2:** *Panchamahabhuta*, their possible physical attributes, modality and organ of perception

*Mahabhuta*	Physical attributes	Modality of perception	Organ of perception
*Akasha*	State of void	*Shabda* (Hearing)	Ear
*Vayu*	State of gas	*Sparsha* (Touch)	Skin
*Agni*	State of energy	*Rupa* (Vision)	Eye
*Jala*	State of liquid	*Rasa* (Taste)	Tongue
*Prithvi*	State of solid	*Gandha* (Smell)	Nose

Modern science supports the proposition that five *Tanmatra* are the progenitors of their visible counterparts by describing basic formative particles (electron, proton and neutron- which could be the *tanmatras*) combining in different proportions and forming atoms, elements and compounds. Ayurveda says *Sarvam dravyam hi panchabhauticam* 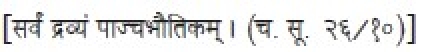 or every substance in the universe is composed of five basic elements only. This further shows a conceptual similarity between modern science and Ayurveda with reference to the idea of substance generation.

The theory of *Tridosha* (three *doshas* namely *Vata, Pitta* and *Kapha*) fundamentally brings *Mahabhuta* theory into a practical, usable and understandable format for its application to the cause of human health. It forms the basis of Ayurvedic physiology and subsequently paves the way to its clinical application. *Tridosha* symbolizes the physico-biological properties of compounds made through a differential combination of *Mahabhutas. Tridosha*, therefore, represents the physiological functioning of a living body, which eventually is the property of its component material. Ayurvedic idea of disease and health is conceived around this concept by identifying a balance of *Tridosha* and eventually that of the *Panchamahabhuta* representing health. An imbalance of *Mahabhuta,* therefore leads to qualitative disturbance of physiological functions subsequently identified as a disharmony of *dosha* causing a disease.

*Panchamahabhuta* are also held responsible for the creation of nonliving things in the same way as they are to the living ones. *Sarvam dravyam hi panchabhauticam* 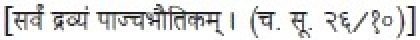 is this generalization of Ayurveda about material composition, which lays the foundation to its understanding that every substance on earth has a potential as medicine (*Nanaushadhibhutam jagat kinchit*).

## PRAKRITI: THE PROTO TYPE

*Prakriti* represents another interesting facet of basic principles of Ayurveda having great impacton predictive medicine. *Prakriti* is described to be formed of characteristic physiological, physical and mental features of an individual, and is classified into subgroups depending on specific *dosha* predominance. Etymologically *Prakriti* (*pra = *primary or first, *kriti = *formation or creation) stands for the prototype representing the basic formative distinction in a given individual. For practical purposes, it can be characterized by the observation of *dosha* activity in the body and eventually to the *Panchamahabhuta* status in the body. Tracing the origin of *Prakriti* to its *Panchbhautic* ancestry one is able to identify certain pattern of combinations of the *mahabhutas*. Ayurveda identifies these patterns of *Prakriti* as subgroups with the predominance of one or more *dosha* in an individual case. Seven subgroups of *Prakriti* are possible representing a differential combination or equi-presence of each one of the *Tridoshas,* namely *Vata, Pitta* and *Kapha.*

Knowledge of the basic *prakriti* of a person is useful tostay in a state of positive health and prevent disease. Recent research has tried to identify the inheritance possibilities of human *Prakriti* by observing positive correlations between specific alleles and *Prakriti* sub-types.[10] An advancement to this correlation is further made by identification of biochemical correlates and whole genome expression to the various *Prakriti* types.[11] A correlation between CYP2C19 genotype and *Prakriti* with fast and slow metabolic features have been attempted recently.[12] Features representing *Prakriti* subtypes as per their *dosha* specification have also been attempted for their statistical validation.[13]

## TRIDOSHA: MODUS OPERANDI OF AN OPEN SYSTEM

*Tridosha* is the physiological basis around which practical Ayurveda revolves. The theory of *Tridosha* is developed primarily as a tool to quantify the pathogenesis and consequently to quantify the need of healthcare interventions in any given condition. As it is practically difficult to assess the status of *ahabhutas* whether in a state of balance or not to predict healthy state or diseased state) assessment of the status of the *tridoshas* has become come the preferred way of decision-making in therapeutics in Ayurveda. Three basic functions operating through a constant interplay between the environment and the individual are thought to be required to maintain the integrity of a living system. Hankey's (2007) proposal of input–output, throughput and storage as three basic functions of an open system resemble functions of *Vata*, *Pitta* and *Kapha*, respectively, as proposed in Ayurveda.[14] The functions of input–output, throughput and storage or incidentally of *Vata*, *Pitta* and *Kapha* are primary requisites for the existence of any living system. In turn, these are considered to be the manifestation of compositional complexity of a person. Consequently, a *Mahabhautic* root to the *Vata*, *Pitta* and *Kapha* is identified as described in Table 3. By observing the function of their representative *dosha*, a deficit or the excess of the *Mahabhuta* can be identified as the cause behind the state of sickness or health.

**Table 3:** *Mahabhuta* and their representative *dosha*

Predominant *Mahabhuta*	Representative *Dosha*
*Akash + Vayu*	*Vata*
*Agni*	*Pitta*
*Jala + Prithvi*	*Kapha*

## *RASA:* A TOOL TO IDENTIFY AND QUANTIFY APPROPRIATE MEASURE OF HEALTH MAINTANANCE.

Pathology, as proposed in Ayurveda, is the manifestation of *Mahabhautic* imbalance leading to physiological disturbances of *Vata*, *Pitta* or *Kapha* resulting intheir disequilibrium. Identification of a *dosha* disharmony in terms of its deficit, excess or qualitative disturbance and consequently of *Mahabhuta*, and then its re-correction to its original state is requited to restore health.

Through the *nanaushadhibhutam jagat kinchit* 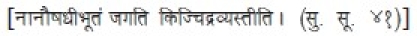 dictum, Ayurveda identifies every object in the universe as a potential medicine based upon its *Panchbhautic* composition. The *panchabhautic* characteristic of medicinal materials used in Ayurveda (herbs and minerals) cannot be identified using the conventional *Tridosha* theory.

Thus, other perceptible, yet scientific and representative parameters have been described for identification of their elemental predominance. *Rasa* (taste) is conceived as a perceptible and representative attribute of a substance which reflects reproducibly and accurately its elemental composition. Ayurveda has described six *rasas* and has proposed a clear correlation of *Rasa* to the elemental predominance in substances [Table 4].[15]

**Table 4:** *Rasa* and their elemental composition

*Rasa*	Elemental composition
*Madhura*	*Jala, Prithvi*
*Amla*	*Prithvi, Agni*
*Lavana*	*Jala, Agni*
*Katu*	*Vayu Agni*
*Tikta*	*Vayu, Akasha*
*Kashaya*	*Vayu, Prithvi*

The idea of *Rasa* as an attribute representing pharmacological properties of a substance has recently been brought under a scientific scrutiny.[16] Ibuprofen and Oleocanthal are found to have similar pharmacological action despite obvious differences in their chemical structures. These substances produce a strong stinging sensation in the throat, and irrespective of their structural differences are found to have COX-1 and COX -2 inhibiting properties.[17] Their similarity of action is attributed to their taste and not to their chemical structure. *Rasa*, therefore, acts as an intermediary between physician and patient for a logical identification of right medicine in individual clinical conditions where a diagnosis in terms of elemental imbalance is made.

Besides *Rasa*, Ayurveda further identifies a few more attributes to determine pharmacological property of a substance. These include *Guna* (property), *Virya* (potency), *Vipaka* (post- digestion property) and *Prabhava* (special effect) and all these are needed to fully understand the complete pharmacological effect of a substance. The *Rasa* and corresponding attributes of a substance, independently or in coherence are used in Ayurveda for identifying the pharmacological effect and subsequently for the clinical usage of a medicine. Different *Rasa* have also been described to produce variable effects on functions of *dosha.* This makes the consideration of *dosha* and *rasa* of a medicine very crucial in the practice Ayurvedic medicine [Table 5].

**Table 5:** *Rasa* and their effect upon *dosha*

*Rasa*	Effect on *dosha*
*Madhur*	*Pro Kapha, Anti Vata, Anti Pitta*
*Amla*	*Pro kapha, Anti Vata, Pro Pitta*
*Lavana*	*Pro kapha, Anti Vata, Pro Pitta*
*Katu*	*Pro Vata, Anti Kapha, Pro Pitta*
*Tikta*	*Pro Vata, Anti Kapha, Anti Pitta*
*Kashaya*	*Pro Vata, Anti Kapha, Anti Pitta*

## SCIENCE AND AYURVEDA: REDISCOVERING THE LOST KEYS

The 21st century began with a few landmark observations that helped decisively to rediscover the lost links between modern Science and Ayurveda. This period has also proposed certain new models to comprehend Ayurvedic fundamental tenetson grounds acceptable to the Western world.[14] The evolved and explicit human physiology and behavioral science have been described to have their seeds in the philosophy of Ayurveda.[7] The identification of a genomic link to the theory of *Prakriti* led to a search for possible classification of people on their *Prakriti* based on theirgenetic make up.[10] These studies could eventually lead to a personalization of medical practice on the basis of *prakriti* as is conceived in Ayurveda.[18]

Reappraisal of Ayurvedic phytochemistry gives a strong support to the Ayurvedic fundamental constructs about the taste (*Rasa*), after taste (*Vipaka*), special effects (*Prabhava*) and pharmacological impacts (*Guna*) of medicinal plants.[16] Ayurvedic pharmaceutics are receiving a new thrust through a reappraisal of *Bhasma* preparations (preparations, where herbs, minerals and metals are incinerated to ash under supervised conditions) as novel nano-technological applications. Typical features of Ayurvedic *Bhasma* have been recently demonstrated through transmission electron microscopy and atomic force microscopy.[19] A further study has shown *Swarna* (Gold) *bhasma* principally constituted of globular gold particle of 56–57 nm. Interestingly, the same study also revealed *Swarna bhasma* to be devoid of any other heavy metal or organic material by its screening through Atomic Absorption Spectroscopy (AAS) and Infrared Spectroscopy (IS).[19] This study also put to rest concerns about the presence of heavy metals in ayurvedic preparations which otherwise clouds the popular use of Ayurvedic medicines abroad.[20] The nano-particle size of ayurvedic *Bhasmas*, has been confirmed in another study,[21] where it is proposed that the nano-particles are responsible for its fast and targeted action. These nano-particles are proposed to be delivered to the target through rapid cellular internalization. Subsequent actions upon DNA/RNA molecule and protein synthesis within the cell are further hypothesized as possible mechanisms for rapid onset of therapeutic actions of *Bhasma* preparations. Pyrgiotakis (2007), with the help of Raman spectroscopy, has demonstrated the effect of *Yashada* (Zinc) *bhasma* on intracellular DNA and proteins of the treated human lung adenocarcinoma cell line (A549).[22] Another study found gold nano-particles (4 nm size) helped in increased apoptosis in B-Chronic Lymphocytic Leukemia (CLL). Incidentally, CLL is an otherwise incurable disease predominantly characterized by resistance to apoptosis.[23] It is observed that the nano- medical application of various drugs is proportionate to their particle size and shape. Smaller the particle, the quicker is the cellular internalization and consequent effects. It is interesting to reiterate here that the pharmacological efficacy of a *Bhasma* preparation is largely attributed to the number and type of *Puta* (traditional incineration process) used in its making. Increased incinerations, therefore, are able to reduce particle size and subsequently give rise to increased efficacy to a given *Bhasma*.[24]

The toxicity of certain Ayurvedic formulations has become a potential limiting factor towards its globalization. Ayurvedic classic texta have taken a serious note of the potential toxicity of certain herbs, minerals and metals. A strict GMP observation is recommended to overcome inherent toxicity of these preparations. Traditionally, ayurvedic drugs are purified through *Shodhana* (biopurification), which are proposed to reduce drug toxicities through manual, physical or organic ways. Efficacy of *Shodhana* methods in reducing toxicity and enhancing safety of ayurvedic preparations was tested as early as in 1981 when Singh *et al*. demonstrated an improved pharmacological property and reduced toxicity in a *Shodhana* treated *Vatsanabha* (aconite) sample.[25] Throat and Dahanukar (1991) further elaborated this observation by comparing various traditional methods of *Shodhana* for their relative efficacy. In this study, Aconite processed with cow urine was found more effective and less toxic compared to other methods of *Shodhana*.[26] Unfortunately, these studies remained unnoticed for long and hence could not serve a support to establish a scientific basis to ayurvedic pharmaceutics.

Ayurveda also presents some unique clinical applications of its fundamental concepts. *Rasayana* is one such concept having an extensive potential applications. *Rasayana* drugs are described to have anti-aging effects. *Labhopayo hi shastanam rasadinam rasayanam*, [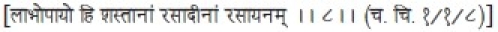] is statement from the Charaka Samhita (*Chikitsa sthana i/i/8*) that says that *Rasayana*,are agents thatare supportive to the qualitative improvement of tissues. A qualitative improvement here essentially refers to the functional and constitutional specifications of a tissue altered by age. Such an enhanced tissue would have optimal functions and also withstand premature aging consequent to its impaired quality. Therefore, anti-aging seems to be the secondary outcome of the *Rasayana*, subsequent to qualitative up-gradation and stabilization of cellswhich is its primary function. Sharangdhara's statement *rasayanam cha tajghyeyam yajjaravyadhi vinashanam* [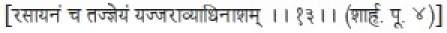] is more apt for its description of the use of *Rasayanas* in *Jaravyadhi* (progeria), *Jara* (aging) and *Vyadhi* (disease).

It is for this reason that, *Rasayana* drugs are said to produce their beneficial effect more promptly in conditions where a suboptimal quality of tissue is leading to its premature aging and suboptimal functioning. In healthy people where the tissue aging is in connotation to the physical age, *Rasayana* can offer only a little to help.

*Rasayana* drugs are proposed to promote tissue longevity through some more novel mechanisms likeReduction of toxin/ metabolic waste load within the cell through their reduced production or increased scavenging, ensuring efficient use of energy within the cell, thus requiring less substrate consumption leading to reduced energy requirement and reduced waste production, initiation of micro-repair by providing essential nutrients by participating in regeneration directly or through promotion of latent enzyme systems.[27]

The studies of Devasagayam *et al*. have added to the understanding of antioxidant action of a few *Rasayana* herbs of Ayurveda. The mechanism of action proposed for these herbs include suppression of free radical formation, break- chain initiation, break-chain propagation, reconstitution of membrane and repair damage.[28]

An approach to understand Ayurvedic pharmacology within a molecular perspective is the recent segment of interfacing between Ayurveda and molecular science. Reserpine, the active alkaloid of *Sarpagandha* (*Rouwolfia serpentina*), till today is the only molecule that blocks vesicular monoamine transporters (VMAT). This generalized blockage exposes biogenic amines to degradation by monoamine oxidize (MAO) leading to their depleted levels. This recent understanding of reserpine uniquely explains the classical indication of *Sarpagandha* for *Unmad* (mania) patients and also explains how its use may lead to biogenic amine depletion and subsequently to depressive episodes.

Ayurveda is often sought as a therapy for cancer patients. There are several common features between the ayurvedic concept of cancer and modern science. Anti-cancer medicines currently used inactivate or activate specific molecules or cell signaling pathways. Within the last three decades, cancer causing genes called oncogenes, cancer-suppressing genes (tumor suppressor genes), cancer growth factors (such as epidermal growth factor and vascular endothelial growth factor), cancer-promoting enzymes (such as cyclooxygenase [COX]-2, matrix metalloproteinase 9, inducible nitric oxide synthetase) and cancer-causing protein kinases (AKT, mitogen- activated protein kinase [MAPK], protein kinase C) have been identified as targets. Although these targets were not known 5000 years ago, the components of herbs used at that time now appear to target these molecules. For instance, nuclear factor kB, which has been known to play a major role in tumorigenesis, is targeted by the components of several herbal remedies described in Ayurveda. Similarly; several herbs have been described in Ayurveda that can suppress either expression of COX-2 or its activity.[29]

There are several more postulates and sporadic works, which strongly present Ayurveda in a scientific and understandable manner to a receptive mind. Thus it is felt that a reappraisal of Ayurveda in light of fundamental science and its advances would be immensely helpful to perceive Ayurveda in true scientific fervor.This article discussed a few important leads from basics of Ayurveda in light of their possible scientific correlates to reappraise them in tune to contemporary knowledge.

## CONCLUSION

The very core of Ayurveda is formed from some very basic concepts e.g. panchabhautic theory, the prakriti concept which is used to describe the predisposition to and prognosis of disease as well as governs the choice of the therapy, balance and imbalance of the three dosha (vata, pitta and kapha) in the development of disease. Interestingly, Ayurveda further expands on these theories to plan interventions that would correct the imbalance. The actions of medicines are described through their various properties (like rasa, guna, veerya, vipaka,and prabhava) based inherently on their elemental composition. It is the need of the hour to use modern technology to explore the relevance of these concepts, so that they may be interpreted in light of contemporary scientific language to offer modern health care.
